# Poster Session I - A34 IMAGING MASS CYTOMETRY REVEALS ALTERED IMMUNE CELL SPATIAL RELATIONSHIPS DURING INFLAMMATORY BOWEL DISEASE EXACERBATIONS

**DOI:** 10.1093/jcag/gwaf042.034

**Published:** 2026-02-13

**Authors:** O Sienkiewicz, D Mulder

**Affiliations:** Queen’s University, Kingston, ON, Canada; Queen’s University, Kingston, ON, Canada

## Abstract

**Background:**

Inflammatory Bowel Disease (IBD) is a chronic, relapsing and remitting autoimmune disease with growing prevalence in North America. It presents with a distinct spatial element, with many patients only having inflammation in specific regions of their gut, with other regions being unaffected. Cellular organization in the multi-layered tissue levels is crucial, with epithelium, lamina propria, and muscularis each hosting different cell populations required to maintain successful barrier function.

**Aims:**

To gain insight into how spatial relations between immune cells differ between inflamed and uninflamed tissue sections, we performed imaging mass cytometry (IMC) on inflamed and uninflamed colon and ileal tissue biopsies collected from IBD patients.

**Methods:**

Biopsies from 15 histologically active (case) and 15 histologically inactive (control) IBD patients were pair matched based on age, sex, disease subtype, and sample region. IMC was performed using a uniquely developed panel of 33 markers designed to identify structural and immune cells. Cells were segmented using a deep learning model. Using an unsupervised clustering approach, we phenotyped 324,786 cells based on marker expression. We generated 17 clusters of cell types of immune and structural origin. Interaction analysis was performed based on gut location and inflammation status to determine which cells are found in relation to one another.

**Results:**

In both gut regions, spatial interactions of classical macrophages (CD169+) and M2 macrophages (CD206+) differed depending on inflammation status. In uninflamed tissue, CD4+ T cells, CD8+ T cells, mast cells, plasma cells, and proliferating leukocytes are predominantly associated with M2 macrophages, however in inflamed tissue classical macrophages became predominantly associated with these immune cell types.

**Conclusions:**

Spatial analysis revealed unique cellular interaction patterns dependent on disease exacerbation and gut location, highlighting potential cellular relationships involved in IBD homeostasis. In both the colon and ileum, macrophages strongly interact with other innate and adaptive immune cells. Whether this phenotypical shift is due to in-tissue proliferation of macrophages or recruitment and polarization of blood monocytes is an important distinction which warrants further investigation.

**Funding Agencies:**

None

